# Antibody and Plasmablast Response to 13-Valent Pneumococcal Conjugate Vaccine in Chronic Lymphocytic Leukemia Patients – Preliminary Report

**DOI:** 10.1371/journal.pone.0114966

**Published:** 2014-12-15

**Authors:** Marcin Pasiarski, Jacek Rolinski, Ewelina Grywalska, Agnieszka Stelmach-Goldys, Izabela Korona-Glowniak, Stanislaw Gozdz, Iwona Hus, Anna Malm

**Affiliations:** 1 Department of Hematology, Holycross Cancer Center, Kielce, Poland; 2 Department of Clinical Immunology and Immunotherapy, Medical University of Lublin, Lublin, Poland; 3 St. John’s Cancer Center, Lublin, Poland; 4 Department of Chemotherapy and Clinical Oncology, Holycross Cancer Center, Kielce, Poland; 5 Faculty of Health Sciences, Jan Kochanowski University, Kielce, Poland; 6 Department of Clinical Transplantology, Medical University of Lublin, Lublin, Poland; 7 Department of Pharmaceutical Microbiology, Medical University of Lublin, Lublin, Poland; Centers for Disease Control & Prevention, United States of America

## Abstract

**Background:**

Chronic lymphocytic leukemia (CLL) leads to significant immune system dysfunction. The predominant clinical presentation in 50% of patients involves recurrent, often severe, infections. Infections are also the most common (60–80%) cause of deaths in CLL patients. The scope of infections varies with the clinical stage of the disease. Treatment-naive patients typically present with respiratory tract infections caused by encapsulated bacteria *Streptococcus pneumoniae* and *Haemophilus influenzae*. Since 2012, the 13-valent pneumococcal conjugate vaccine (PCV13) has been recommended in the United States and some EU countries for pneumococcal infection prevention in patients with CLL (besides the long-standing standard, 23-valent pneumococcal polysaccharide vaccine, PPV23). The aim of this study was to compare the immune response to PCV13 in 24 previously untreated CLL patients and healthy subjects.

**Methods:**

Both groups were evaluated for: the levels of specific pneumococcal antibodies, the levels of IgG and IgG subclasses and selected peripheral blood lymphocyte subpopulations including the frequency of plasmablasts before and after immunization.

**Results:**

Adequate response to vaccination, defined as an at least two-fold increase in specific pneumococcal antibody titers versus pre-vaccination baseline titers, was found in 58.3% of CLL patients and 100% of healthy subjects. Both the CLL group and the control group demonstrated a statistically significant increase in the IgG2 subclass levels following vaccination (P = 0.0301). After vaccination, the frequency of plasmablasts was significantly lower (P<0.0001) in CLL patients in comparison to that in controls. Patients who responded to vaccination had lower clinical stage of CLL as well as higher total IgG, and IgG2 subclass levels. No significant vaccine-related side effects were observed.

**Conclusions:**

PCV13 vaccination in CLL patients is safe and induces an effective immune response in a considerable proportion of patients. To achieve an optimal vaccination response, the administration of PCV13 is recommended as soon as possible following CLL diagnosis.

## Introduction

Chronic lymphocytic leukemia (CLL) is the most common type of leukemia in European and North American adult populations leading to significant immune system dysfunction [1]. The dominant clinical presentation in 50% patients are recurrent, often severe, infections [2]–[4]. Infections are the most common cause of deaths in CLL patients, with 60–80% mortality [4], [5]. Most patients with early-stage of CLL do not need any treatment when the disease is first diagnosed because early treatment offers no survival advantage [6]. Only patients with active disease require therapy [7]. The first-line treatment recommended for CLL patients in good general condition with no significant comorbidities is immunochemotherapy with purine analogue fludarabine combined with cyclophosphamide and rituximab (FCR regimen) [8]. In patients in poorer general health, burdened with comorbidities, chlorambucil or bendamustine might be used in monotherapy or combined with rituximab [9]. Purine analogues result in quantitative and qualitative T-cell abnormalities that can persist for up to 2 years [10] and rituximab results in hypogammaglobulinemia. The therapy-induced immunosuppression further increases the risk of serious infections, that supports the conception to vaccinate the CLL patients as soon as possible after disease diagnosis.

There are a number of factors causing immune suppression in CLL patients. They include both the specific (humoral and cellular) as well as innate immunity (dysfunction of the complement system, NK-cells, neutrophils and monocytes). In the case of specific immunity, the key factors are: hypogammaglobulinemia due to B-cell and T-cell dysfunction. Hypogammaglobulinemia typically involves all classes of immunoglobulins (IgG, IgA, IgM), but mostly the IgM class (in 56.7% of patients) as well as IgG3 (52%) and IgG1 (28%) subclasses [11], [12]. Immunoglobulin deficiency can be found in nearly all patients, with only 17% of patients having normal levels of all immunoglobulin classes and subclasses (IgG1, IgG2, IgG3, IgG4). The incidence of hypogammaglobulinemia increases as the disease progresses, reaching 100% in patients with Binet stage C. Hypogammaglobulinemia may be present already at CLL diagnosis [11]. Some causes of infection, apart from the developing disease itself, may also be associated with the more advanced age of the patients, frequent comorbidities (such as diabetes mellitus, heart failure) or treatment-induced immune suppression.

The pathogens responsible for infections in CLL patients are mostly bacteria (67%), sometimes viruses (25%), and occasionally fungi (7%) [13]–[15]. The scope of infections varies with the clinical stage of the disease. Treatment-naive patients typically present with respiratory tract infections caused by the encapsulated bacteria *Streptococcus pneumoniae* and *Haemophilus influenzae*, as well as urinary tract infections caused by *Escherichia coli* [13], [15]. An inverse correlation between IgG levels and the incidence and severity of infections, especially those caused by *Haemophilus influenzae* and *Streptococcus pneumoniae,* has been demonstrated [16].

In the US and in many EU countries, *Streptococcus pneumoniae* vaccinations are recommended in immunocompromised patients, including patients with CLL. For many years now, the 23-valent pneumococcal polysaccharide vaccine (PPV23) has been used in CLL patients; however, the resulting immune response has been poor, with only 20–25% patients achieving a two-fold increase in antibody titres versus baseline [17]–[20]. Since 2012, both the 13-valent pneumococcal conjugate vaccine (PCV13) and PPV23 have been recommended for pneumococcal infection prevention in patients with CLL. The effectiveness of PCV13 in CLL patients has not been assessed.

The objective of the study was to assess the immune response to PCV13 in 24 treatment-naive CLL patients versus healthy subjects. Both groups were evaluated for: the levels of specific pneumococcal antibodies, the levels of IgG and IgG subclasses, selected peripheral blood lymphocyte subpopulations including the proportion of plasmablasts before and after immunization.

## Methods

### The Study Group and the Control Group

A total of 24 previously untreated patients with CLL who were diagnosed in the Hematology Department at Holycross Cancer Centre in Kielce were included in the study. Table 1 (a) presents the characteristics of the study and control groups. All patients enrolled in the study were in the stage 0–II according to Rai classification [21]. None of the patients had been receiving drugs affecting the immune system, none showed any signs of infection (at least 2 months prior to the study), or shown any signs of autoimmune or allergic disease and none had received blood transfusions. The control group consisted of 15 healthy, age- and sex-matched individuals - Table 1 (a).

**Table 1:** (a) Characteristics of CLL patients and control group. (b) Percentages of plasmablasts and serum anti-pneumococcal antibody as well as IgG2 levels before and after PCV13 vaccination in the CLL patients and control group.

(a)											
Parameter				CLL patients (n = 24)			Control group (n = 15)				
Gender	Females			54%			44%				
	Males			46%			56%				
Age	mean ± SD			66±7.9			68.6±8.21				
	median (min–max)			66 (47–79)			68.5 (54–83)				
Rai stage	0			50%			Not applicable				
	I			16.7%			Not applicable				
	II			33.3%			Not applicable				
Lymphocytosis	mean ± SD			29.087±24.922			2.044±600.88				
	median (min–max)			23.460 (5.321–10.9570)			1.995 (1.130–3.310)				
LDH level (U/L)	mean ± SD			345±76.35			343.81±50.07				
	median (min–max)			332 (202–570)			347.5 (251–435)				
Β2microglobulin levels (µg/L)	mean ± SD			2.163.5±572.48			1.870.69±339.56				
	median (min–max)			2.139.5 (1.397–3.408)			1.957 (1.178–2.285)				
IgG level (g/L)	mean ± SD			9.08±2.27			10.06±1.71				
	median (min–max)			9.06 (3.27–12.87)			10.15 (7.23–14.26)				
**(b)**											
Parameter	N	Median	Lower quartile			Upper quartile			p		
**CLL patients**											
CD19+/IgD−/CD27++ cells [%]											
-before vaccination	24	0.03	0.01		0.06			0.029			
-after vaccination	24	0.03	0.02			0.13					
Anti-pneumococcal IgG antibodies [mU/mL]											
-before vaccination	24	373.40	240.05			767.65			<0.0001		
-after vaccination	24	1401.65	568.50			3114.50					
Level of IgG2 [g/L]											
-before vaccination	24	2.90	2.14			3.63			0.012		
-after vaccination	24	2.92	2.11			3.73					
**Control group**											
CD19+/IgD−/CD27++ cells [%]											
-before vaccination	15	0.26	0.20			0.50			<0.0001		
-after vaccination	15	6.95	2.71			12.85					
Anti-pneumococcal IgG antibodies [mU/mL]											
-before vaccination	15	297.35	152.00			664.80			<0.0001		
-after vaccination	15	3197.05	1482.10			3931.70					
Level of IgG2 [g/L]											
-before vaccination	15	2.92	2.52			4.29			0.009		
-after vaccination	15	3.42	2.99			4.25					

CLL was diagnosed based on the National Cancer Institute (NCI) International Workshop on CLL (IWCLL) guidelines [7], [22]. First patient received PCV13 in June 2012, and last one – in January 2013. The mean follow-up period from the time of vaccination was 21.02±3.37 months (median: 20.75 months, minimum: 18 months, maximum: 24 months). The complete blood count, beta-2-microglobulin, and lactate dehydrogenase (LDH) serum concentration, as well as imaging examinations were conducted with the use of standard diagnostic and radiological laboratory methods. Five patients (20.8%) had hypogammaglobulinemia (IgG <7 g/L). During the study, no patient developed an infection detectable with a routine physical examination. None of the patients enrolled in the study died and 22 persons (91.67%) still do not require treatment.

All study subjects gave their written consent for participation. The study protocol was approved by the Bioethics Committee of the Regional Chamber of Physicians in Kielce (No. KB7/2012).

The peripheral blood samples were drawn from the basilic vein for the following tests: 1) serum specific pneumococcal antibody titers before vaccination (3 mL of peripheral blood collected to tubes with a clotting activator) and 30 days after vaccination (3 mL of peripheral blood collected to tubes with a clotting factor), 2) percentage of plasmablasts, defined as CD19+/IgD−/CD27++ before vaccination (5 mL of peripheral blood collected to tubes with the anticoagulant EDTA) and 7 days after vaccination (5 mL of peripheral blood collected to tubes with the anticoagulant EDTA), 3) serum total IgG as well as IgG1, IgG2, IgG3, IgG4 levels before vaccination (5 mL of peripheral blood collected to tubes with a clotting activator) and 30 days after vaccination (5 mL of peripheral blood collected to tubes with a clotting factor). Serum samples were stored at –70°C until the time of specific pneumococcal antibody titers analysis. Percentages of plasmablasts were assessed on fresh peripheral blood samples from CLL patients and healthy volunteers. Serum total IgG as well as IgG1, IgG2, IgG3, IgG4 levels were measured in fresh serum samples.

### Vaccine

Immunization of CLL patients and controls was conducted with the use of 13-valent conjugate vaccine Prevenar13 (Pfizer), containing polysaccharide antigens of the following pneumococcal serotypes: 1, 3, 4, 5, 6A, 6B, 7F, 9V, 14, 18C, 19A, 19F, 23F, conjugated with the carrier protein CRM197. The vaccine was injected via the intramuscular route. A single dose of PCV13 was administered. None of the patients and controls received previous PSV23 vaccination.

### Plasmablast Evaluation

The peripheral blood samples were diluted with 0.9% calcium (Ca^2+^) and magnesium (Mg^2+^)-free phosphate buffered saline (Biochrome AG, Germany) at a 1∶1 ratio. The diluted material was layered onto 3 mL Gradisol L (Aqua Medica, Poland) with specific gravity of 1.077 g/mL, and then centrifuged in density gradient at 700 g for 20 minutes. The obtained peripheral blood mononuclear cells (PBMCs) were collected with Pasteur pipettes and washed twice with Ca^2+^Mg^2+^-free PBS for 5 minutes. Subsequently, the cells were suspended in 1 mL Ca^2+^Mg^2+^-free PBS, counted with a Neubauer chamber, and their vitality was assessed with trypan blue (0.4% Trypan Blue Solution, Sigma Aldrich, Germany). Following density gradient centrifugation, the PBMCs underwent two- or three-color labelling with adequate quantities of monoclonal antibodies, according to manufacturer’s instructions. The isolated suspension of cells was divided into individual test tubes at 1×10^6^/sample and incubated with monoclonal antibodies. 20 µL of antibodies was added to each sample of evaluated cells and incubated at room temperature for 20 minutes. Following incubation, the cells were washed twice with PBS (at 700 g, 5 minutes) and immediately analyzed with the FACSCalibur flow cytometer (Becton Dickinson, US), equipped with argon laser of 488 nm wavelength. Data acquisition was conducted with specialist FACS Diva Software 6.1.3 system (Becton Dickinson, US). The data were analyzed with CellQuest Pro (Becton Dickinson, US). CaliBRITE (Becton Dickinson, US) was the calibrating system used to optimize flow cytometer settings. For each sample, data was acquired by routine collection of 30,000 events in the lymphocyte gate (region R1) in a forward-scatter (FSC)/side-scatter (SSC) dot plot. This allowed to exclude erythrocytes, platelets, dead cells, and cell fragments from analysis. Labelled cells were recorded based on the created lymphocyte gate. The results were presented as percentage of CD45+ cells stained with antibody. The following monoclonal antibodies, conjugated with their appropriate fluorochromes, were used: FITC-mouse anti-human IgM (catalogue No. 555782), FITC-mouse anti-human IgD (catalogue No. 555778), PE-mouse anti-human CD19 (catalogue No. 555413), PE-mouse anti-human CD38 (catalogue No. 555460), PE-Cy 5-mouse anti-human CD19 (catalogue No. 555414), and APC-mouse anti-human CD27 (catalogue No. 337169) (Becton Dickinson, US). In order to detect the non-specific bindings, the mouse isotype controls were used: FITC Mouse IgG1 κ, Isotype Control, Clone MOPC-21 (catalogue No. 555748), FITC Mouse IgG2a κ, Isotype Control, Clone G155-178 (catalogue No. 555573), PE Mouse IgG1 κ, Isotype Control, Clone MOPC-21 (catalogue No. 555749), and APC Mouse IgG1 κ, Isotype Control, Clone MOPC-21 (catalogue No. 550854), respectively. The proportion of plasmablasts in peripheral blood was evaluated on the vaccination day (prior to vaccination) and 7 days after vaccination.

### Serum Pneumococcal Antibody Assessment

The serum pneumococcal antibody assessment was performed in all subjects before vaccination (on the vaccination day) and 30 days after vaccination. The amount of anti-capsular-polysaccharides antibody specific for 23 pneumococcal serotypes (1, 2, 3, 4, 5, 6B, 7F, 8, 9N, 9V, 10A, 11A, 12F, 14, 15B, 17F, 18C, 19A, 19F, 20, 22F, 23F, 33F) was determined using a commercial ELISA test (ELIZEN Pneumococcus IgG Assay, Zentech, Belgium). Each of the serum samples were pre-adsorbed with 10 µg/ml polysaccharide C (C-PS, Statens Serum Institute, Denmark) for 1 hour at 37°C before quantification to increase the specificity of the test [23]. The evaluation procedure was followed according to manufacturer’s instructions and an automatic VICTOR^3^ reader (Perkin Elmer, US) was used for result interpretation.

### Assessment of IgG Subclasses

The assessment of IgG subclasses: IgG1, IgG2, IgG3, and IgG4 was performed via nephelometric techniques with nephelometer BN2 (Dade Behring, Germany), according to manufacturer’s instructions.

### Other Parameters

Routine immunophenotyping of peripheral blood lymphocytes, β_2_microglobulin, IL-6, complement components 3 and 4 (C3 and C4), LDH, IgG, IgM, IgA serum levels, and chromosome abnormalities: del11q22.3-q23.1, chromosome 12 trisomy, del13q14-23.1, del17p13.1 were measured in all CLL patients. Complete blood count tests and measurements of serum IgA, IgM, IgG, IgG1, IgG2, IgG3, IgG4 levels, were conducted before, and 1 month after vaccination.

### Statistical Analysis

Normal distribution of continuous variables was verified with Shapiro-Wilk Test. Statistical characteristics of continuous variables were presented as median, extreme values (minimum and maximum), as well as arithmetic means, and standard deviations (SD). Inter-group comparisons were conducted with the Mann-Whitney U test, as well as a univariate ANOVA with the *post-hoc* Tukey’s test or the Kruskal-Wallis test followed by the *post-hoc* Dunn test. The Pearson’s chi-squared test or Fisher’s exact test were used for inter-group comparison of discontinuous variable distribution. All calculations were conducted with Statistica 10 (StatSoft, US), with the significance level set at P<0.05.

## Results

**Figure 1:**
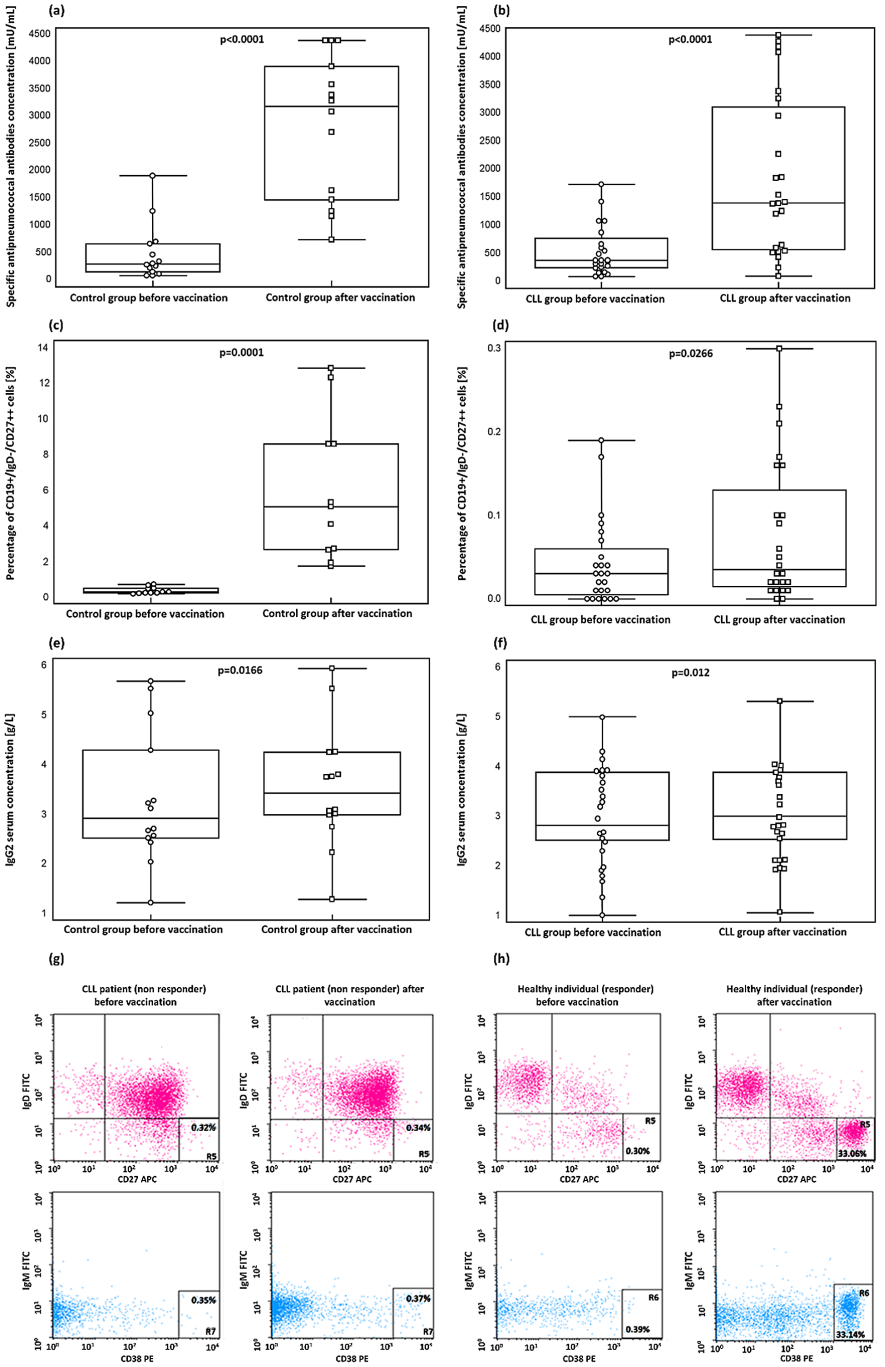
Specific antipneumococcal antibody titers before and after PCV13 vaccination in: (a) the control group and (b) CLL patients (P<0.001, and P<0.001, respectively). Proportion of plasmablasts before and after PCV13 vaccination in: (**c**) the control group and (**d**) CLL patients (P = 0.001, and P = 0.0266, respectively). Serum IgG2 levels before and after PCV13 vaccination in: (**e**) the control group and (**f**) CLL patients (P = 0.0166, and P = 0.012, respectively). A multiparameter flow cytometric analysis of the percentages of plasmablasts before and 7 days after vaccination. Representative example of gating on CD19+ B-cell subpopulations. Percentages of cells in the different quadrants are shown. CD19+/IgD−/CD27++ plasmablasts (upper left quadrant) and CD19+/IgM/CD38++ plasmablasts (lower left quadrant) before vaccination and CD19+/IgD−/CD27++ plasmablasts (upper right quadrant) and CD19+/IgM/CD38++ plasmablasts (lower right quadrant) after vaccination in: (**g**) the individual CLL patient who did not respond to PCV13 administration and (**h**) the individual healthy subject from the control group, who responded to PCV13 administration.

Table 1 (b) presents significant differences in percentage of plasmablasts, serum anti-pneumococcal antibody levels, as well as IgG2 serum levels before and after PCV13 vaccination in the study and control group. Fig. 1 (a–f) presents graphs, illustrating obtained results in the control group and CLL patients.

Adequate response to vaccination was defined as an at least two-fold increase in specific pneumococcal antibody titers versus pre-vaccination baseline titers. This vaccination response criterion was achieved by 100% of subjects from the control group and 58.3% of CLL patients.

Prior to vaccination, there were no significant differences in the specific pneumococcal antibody titers between the CLL group and the control group (P = 0.410).

The specific pneumococcal antibody titers following PCV13 vaccination were significantly higher in the control group versus the CLL group (P = 0.037).

Evaluation of immunoglobulin G subclasses revealed, with a statistically significant increase in IgG2 levels after PCV13 vaccination in CLL patients (P = 0.012).

The frequency of plasmablasts in the CLL patients before vaccination was significantly higher than that in the control group (P<0.001). After vaccination, the percentage of these cells was also significantly lower (P<0.001) in the CLL patients comparing to the control group. The proportion of plasmablasts after vaccination was significantly higher both in the control group and patients with CLL (P = 0.0001, and P = 0.0266, respectively, Fig. 1 (c) and (d)). Fig. 1 (g) and (h) presents an assessment of the proportion of CD19+/IgD−/CD27++ plasmablasts before vaccination (upper left quadrant) and after vaccination (upper right quadrant) and an assessment of the proportion of CD19+/IgM/CD38++ plasmablasts before vaccination (lower left quadrant) and 7 days after vaccination (lower right quadrant) in a CLL patient who did not respond to PCV13 vaccine administration (g) and in a healthy subject from the control group, who responded to PCV13 vaccine administration (h).

Peripheral blood tests showed no significant differences in the absolute lymphocyte count before and after vaccination.

## Discussion

The efficacy of immunization in reduction of infections in patients with CLL was not evaluated in randomized studies. However, lower response to vaccination against pneumococci and the influenza virus was observed in CLL patients comparing to the healthy subjects [24]–[26]. Studies conducted in 2001 showed that polysaccharide vaccines do not ensure adequate efficacy in CLL patients, while conjugated vaccines (against *Haemophilus influenzae* type B and the tetanus toxin) provide better protection [18]. Subsequent studies showed a better response to conjugated vaccines against *Streptococcus pneumoniae* and *Haemophilus influenzae* type B in CLL patients [20], [26].

The approval of the conjugated vaccine PCV13 for adults in 2012 in the EU and several months later in the US raised the hope of achieving an improved response to the vaccination in patients with lymphoid malignancies, including CLL. PPV23 used so far provided only a 20–25% response to immunization [17]–[20]. According to current recommendations for CLL patients PCV13 vaccine should be administered first, followed by PPV23 after at least 8 weeks.

During the course of CLL, hypogammaglobulinemia occurs with the incidence ranging from 10 to 100% depending on the stage and duration of disease [4]. IgG deficiency is especially strongly associated with infections induced by *Streptococcus* and *Haemophilus* [27]. These pathogens most commonly cause pneumonia, but may also lead to serious complications such as bacteremia and septic shock [27], [28]. Studies by Chou et al. showed that pneumococcal pneumonia and bacteremia are common causes of deaths among patients with neoplastic disease, with lymphoproliferative diseases posing a particularly high risk [29]. Natural resistance to *Streptococcus pneumoniae* infections develops during exposure to the microorganism or its antigens. The infected individual produces specific anti-capsular antibodies, mainly of the IgG2 isotype, which play an important role as opsonins in the process of phagocytosis involving multinucleated leukocytes and pulmonary macrophages. Pneumococcal opsonization is a very important stage, as evidenced by inefficient phagocytosis (e.g. asplenia or neutropenia), antibody production disorders (e.g. hypogammaglobulinemia, selective IgG subclass deficiency) or non-specific opsonins (no complement components), which are predisposing factors for pneumococcal infections [30], [31]. Considering severe and potentially life-threatening complications, pneumococcal infection prevention becomes especially important. The efficacy of vaccination in CLL patients is often limited due to the dysfunctions mentioned above. Studies by Griffiths et al. showed that, apart from low IgG levels, it is the simultaneously low level of specific antibodies against polysaccharides of pneumococcal capsule that is an important factor affecting the incidence and severity of infections in some CLL patients [16]. This suggests a potential beneficial effect of immunization with conjugated vaccines targeting *Streptococcus pneumoniae* in this patient population.

The purpose of our study was to assess the antibody and plasmablast response to the 13-valent pneumococcal conjugate vaccine in previously untreated patients with CLL in early stages not requiring anticancer therapy. Response to vaccination, defined as an at least two-fold increase in specific antibody titers versus pre-vaccination baseline titers, was detected in 58.3% of CLL patients. Even though the post-vaccination increase in specific pneumococcal antibody titers in the CLL group was lower than that in the control group, it was still at least two times higher than that achieved in earlier studies with non-conjugated vaccines. This result was also confirmed by the assessment of plasmablasts before and 7 days after vaccination, which is one of the established methods for determining early response to vaccination [32], [33]. The results of this study seem to be particularly relevant for patients with hypogammaglobulinemia, who may exhibit a reduced humoral response. Following PCV13 administration, a statistically significant increase in the proportion of plasmablasts was achieved both in the control group and in the CLL group, which indicates a rapid early response to the vaccine even in the group with CLL-induced immune deficiency. Polysaccharide-protein conjugate vaccines induce thymus-dependent response, which may contribute to a better response in comparison to that after polysaccharide vaccines [26]. The efficacy of immunization with PPV23 is low due to the content of polysaccharides, which – as T-cell-independent antigens – are characterized by lower immunogenicity and which induce no sustained post-vaccination immunity or immunological memory [34]–[36]. Conjugated vaccines are characterized by a higher efficacy, which is even higher in low-grade CLL [20], [37]. In this study we demonstrated a statistically significant post-vaccination increase in IgG2 in CLL patients. Immunoglobulin subclasses, especially IgG2, are also known to be involved in immunity against encapsulated bacteria [38]–[40]. Thus, a serum level of these subclasses is another indicator of PCV13 efficacy. As mentioned before, immune system dysfunction in the course of CLL increases with the duration and clinical stage of the disease. Our studies enrolled patients at the early stages of disease (Rai 0–II) who did not require initiation of chemotherapy. In patients with advanced CLL, immunization program should be adjusted to the treatment schedule, particularly considering treatment with anti-CD20 antibodies that leads to B-cells depletion. Patients with CLL have shown failure in achieving protective levels of antibodies following the flu vaccine if the vaccination was administered just before (2 weeks), during, or up to 6 months after completing chemoimmunotherapy with rituximab [24], [41]. In our study, nearly 60% of CLL patients responded to vaccination. Thus, we can conclude that an optimal way of achieving post-vaccination immunity is to use PCV13 as soon as possible after diagnosis.

Study limitations: we detected a collective titer of 23 vaccine serotypes despite the fact that immunization was conducted with only 13 capsular antigens. One can only suspect that the difference in antibody titers before and after vaccination reflects mainly the antibodies against PCV13 serotypes. In the present study, overall subclass determinations of total antibodies in the serum and no serotype-specific antibodies were measured. However following this preliminary report, we plan to assess IgG1, IgG2, IgG3, and IgG4 serotype-specific antibodies in both CLL patients and control group. Currently, we are analyzing the response to PCV13 with a subsequent administration of PPSV23 eight weeks later. According to CDC guidelines, such immunization schedule should provide an optimal response in immunocompromised patients; however, it has never been studied in CLL patients. Balmer et al. [42] compared results generated by the assay in which the 23-valent polysaccharide vaccine is the antigen to those obtained by a capsular polysaccharide serotype-specific assay that measures IgG antibodies to 9 common serotypes causing invasive disease (1, 4, 5, 6B, 9V, 14, 18C, 19F, 23F). Discrepancies in 45% of the results were observed in a direct comparison between the two assays. In each case a positive titer was obtained on the clinical assay but IgG levels on the serotype-specific assay were below the putative protective level for at least one of the 9 serotypes assayed [42]. Our preliminary data are very interesting/promising but they require confirmation and extending by using WHO recommended an enzyme-linked immunosorbent assay for quantitation of human IgG antibodies specific for *Streptococcus pneumoniae* capsular polysaccharides (Pn PS ELISA) [43]. Our plan is also to present the results of functional antibody assays performed by the ELISPOT method and using opsonophagocytosis assay (OPK); presently we are comparing results obtained following PCV13 and PPSV23 vaccination.

Although our speculation are not supported with clinical data confirming vaccination efficacy in reducing the incidence and mortality associated with pneumococcal infections in CLL patients, immune system stimulation itself (i.e. achieving an increased number of plasmablasts) is an important phenomenon. All vaccinated patients are monitored for the incidence of infections. In vaccinated patients no serious bacterial nor viral infections, despite recurrent infections before immunization, were observed. None of the patients enrolled in the study died and 22 persons (91.67%) still do not require antileukemic treatment. In conclusion, the results of the current study show that PCV13 vaccination in CLL patients is safe and provide clinical benefit inducing an immune response in a considerable proportion of patients. Presented data support the need for vaccination in patients with CLL and to achieve an optimal vaccination response, the administration of PCV13 is recommended as soon as possible following CLL diagnosis.
